# Engagement, Exhaustion, and Perceived Performance of Public Employees Before and During the COVID-19 Crisis

**DOI:** 10.1177/00910260211073154

**Published:** 2022-01-29

**Authors:** David Giauque, Karine Renard, Frédéric Cornu, Yves Emery

**Affiliations:** 1 University of Lausanne, Switzerland

**Keywords:** new ways of working, forced teleworking, well-being, perceived performance, engagement

## Abstract

At the outset of the COVID-19 pandemic, the Swiss federal government implemented a lockdown that prompted a majority of private and public organizations to implement teleworking solutions for their employees. This study aimed to examine the impact of work modalities, job-related, relational, and organizational climate variables on employees’ engagement, exhaustion, and perceived performance both before and during the forced teleworking period. Based on the job demands-resources framework, a survey was conducted (*N* = 1,373) in a Swiss Cantonal public administration. Results show that while the forced telework period positively influenced employees’ work autonomy and work–life balance, it negatively influenced their degree of collaboration and perceived job strain but did not affect their engagement levels. The freedom to organize ones’ own work and collaboration with colleagues were identified as the main resources that positively influence employees’ engagement and perceived performance while limiting exhaustion.

The COVID-19 crisis led governments around the world to impose restrictions to contain the spread of the coronavirus. These restrictions included recommendations or injunctions made to public and private organizations to introduce new ways of working (NWW) and, more specifically, in the Swiss context, to favor remote working or teleworking. In Switzerland, a democratic and federalist system (see Kriesi & Trechsel, 2008), the Swiss Federal Council made the decision on March 16, 2020, to close schools, restaurants, shops, bars, and nightclubs. Federal political authorities decreed that telework was mandatory for all public and private organizations that had the capacity for remote work. These extreme measures, undertaken at the federal level, meant that all Cantons (the 26 regional political entities that exist in Switzerland, and are the institutional equivalent of the states in the United States) were forced to follow these rules until mid-May 2020. However, citizens still had some freedom to leave their homes.

These federalist measures aiming to compel organizations to introduce teleworking led to a shift in working conditions as well as a change in work design and execution. In the case of public organizations, these changes led public servants to work mostly remotely. Teleworking is one component of NWW, which refers to a set of practices that comprise flexibility in working hours, flexibility in the place of work (teleworking, satellite offices, or mobile working), use of new technology networks and collaborative tools, and greater access to knowledge. Teleworking implies that employees work outside their professional office spaces while keeping in touch with colleagues and managers by way of new information and communication technologies (Beauregard et al., 2019).

Therefore, this study aimed to understand the impact on public servants resulting from changes in working conditions owing to teleworking. To do so, this study used the job demands-resources (JD-R) model, which assumes that the job characteristics, namely, job demands and job resources, are important predictors of employee outcomes, such as exhaustion, work engagement, or even perceived performance, in various occupational settings (Bakker & Demerouti, 2017). So far, the empirical evidence regarding the effects of NWW on employee outcomes such as performance, work engagement, satisfaction and health, is mixed and no consensus exists on whether NWW practices have a positive impact on employees’ performance and well-being (Renard et al., 2021). Moreover, to date, in the context of the COVID-19 crisis, there are no empirical data available to assess whether NWW practices exert an influence—positive or negative—on work engagement, work exhaustion, and self-perceived individual performance. To bridge this gap, this study examines public agents’ perceptions of how forced teleworking has impacted their day-to-day activities, work engagement, exhaustion, and perceived performance, while taking into account working conditions before and during the forced teleworking period due to the COVID-19 crisis. To the best of our knowledge, this is the first study that discusses the implications of forced teleworking specifically for public sector employees in the context of the COVID-19 crisis. Accordingly, the main research questions were as follows:

**Research Question 1:** What were the key resources, before and during the forced teleworking period, that positively impacted public sector employees’ engagement and perceived performance while also acting as a buffer against the adverse effects of exhaustion?
**Research Question 2:** What were the constraints (or demands), before and during the forced teleworking period, that negatively impacted public sector employees’ engagement and perceived performance while causing exhaustion?

We attempted to answer these questions using a survey of a sample of 1,367 public sector (response rate: 42.6%) employees working in a Swiss Cantonal administration. This survey allowed us to collect data on public servants’ perceptions of their modalities of work, their job-related characteristics, their work climate (relational aspects and work–life balance) both *before* and *during* the forced teleworking period.

The current study makes multiple contributions to the public administration literature. First, it enhances our understanding of how NWW practices influence employee’s well-being and perceived performance. Second, there has been a call to conduct research to ascertain the influence that organizational- and team-level variables such as organizational culture, organizational climate, and team climate exert on employee engagement. In this respect, the present empirical study takes into consideration two dimensions of work climate and explores their effects on employee outcomes. Third, it helps in identifying the most important antecedents of engagement, exhaustion, and perceived performance of public servants before and during the forced teleworking period. Fourth, by identifying which job demands and job resources positively or negatively influenced employees’ outcomes before and during the period of forced teleworking, this study provides some insights on what organizations should focus on when implementing teleworking practices.

## Theoretical Framework, Literature Review, and Hypotheses

### Theoretical Framework: The JD-R Model

This study’s theoretical framework is based on the JD-R model. It is worth mentioning that the JD-R model draws from several theories, especially job design and job characteristics models of work motivation (Hackman & Oldham, 1976; Karasek, 1979; Wood et al., 2012). Indeed, the JD-R model helps to identify the job characteristics that contribute toward employees’ motivational process or health impairment process. This theoretical perspective is very popular in scientific literature and is relevant for identifying factors that can affect employees’ work engagement, exhaustion, and performance. The advantage of such a theoretical model is that it is adaptable and can include variables that function as resources or demands for the actors. It has been used in various work settings, producing empirically sound results (Bakker & Demerouti, 2007).

The JD-R model categorizes work environments based on two central concepts—job demands and job resources (Bakker et al., 2014). Job demands refer to the physical, social, emotional, cognitive, and organizational dimensions of work that incur physical or psychological costs, while resources include aspects that enable individuals to achieve work objectives, reduce demands and their costs, and engage in personal learning and development. These demands and resources can comprise factors that are work-related (e.g., decision-making latitude, work autonomy, social support, and career opportunities), organization-related (e.g., reorganization and participation in decision-making), or individual-related (e.g., sense of self-efficacy, self-esteem, and optimism).

Job demands and resources lead to two different processes. Job demands are at the root of processes (health impairment processes) that affect the health of employees and can be considered the best predictors of occupational health problems. A central assumption of the JD-R model is that high job demands erode resources of personal energy, leading to emotional exhaustion and job fatigue or exhaustion (Demerouti et al., 2001; Schaufeli & Bakker, 2004). According to empirical studies using the JD-R model to understand the health issues in organizations, work overload, red tape, emotional demands, work–home conflict, and interpersonal conflict are the demand dimensions that lead to stress if they exceed employees’ resources for managing tasks (Giauque et al., 2013; Van den Broeck et al., 2008). However, resources enhance the understanding of motivational processes, which increase job satisfaction, work engagement, and motivation. Work engagement refers to a positive, fulfilling, work-centric state of mind that is characterized by vigor (i.e., high levels of energy and mental resilience while working), dedication (i.e., a sense of significance, enthusiasm, and challenge), and absorption (i.e., being focused and happily engrossed in one’s work). The resources that foster a healthy work atmosphere include job autonomy, opportunities for skill utilization, support from the supervisor and colleagues, financial rewards, career opportunities, team cohesion, harmony, and coaching (Bakker et al., 2014; Beurden et al., 2020; Borst, 2018; Borst et al., 2019; Demerouti et al., 2001). An employee’s performance significantly depends on the interactions between these demands and resources in terms of, for example, turnover, sick leave, work engagement, and job satisfaction.

### Literature Review

Our literature review first considers studies focusing on telework that predate the COVID-19 crisis. Second, it takes a look at studies focusing on forced telework during the COVID-19 crisis. Finally, we turn to the literature related to NWW.

#### Literature on telework before the COVID-19 crisis

Studies focusing on telework were first published in the 1970s (Nilles et al., 1976), and a wide range of disciplines, including management, psychology, sociology, and information systems, took an interest in this area. However, definitions of telework and its components are diverse and numerous (e.g., Baruch, 2001). Nonetheless, scholars tend to agree on two dimensions of telework: being at a distance from the conventional workplace and using information and communication technologies (ICTs) to work (Carillo et al., 2021). A vast body of literature analyses the effects of telework on employees’ performance, health and stress, turnover intentions, and professional isolation. However, there has been no consensus on whether telework is beneficial for or detrimental to employees’ performance and well-being; thus, outcomes of telework have not been clearly identified yet (Beauregard et al., 2019).

A research article published in 2012 (Caillier, 2012) tried to assess the impact of teleworking arrangements on work motivation (satisfaction, organizational commitment, and job involvement) among the employees of a U.S. federal government agency. The findings indicated that employees who were empowered, worked under managers who supported teamwork, had a supportive supervisor, and were confident that their organization was attaining its mission were more likely to report higher levels of work motivation. However, contrary to what was expected by the author, teleworking arrangements were found to be unrelated to work motivation. A more recent empirical study (de Vries et al., 2019) highlighted that there are multiple negative effects of teleworking in the public sector, such as greater professional isolation and less organizational commitment. However, the authors found that teleworking did not affect work engagement, although leader-member exchange was found to be helpful in minimizing the impact of teleworking on professional isolation. Beauregard et al. (2019) reviewed existing research on telework and demonstrated that the outcomes of telework are neither straightforward nor clear. That is, while some studies have reported a positive impact on individual and team-related performance (e.g., Golden & Gajendran, 2019), others have highlighted negative consequences (e.g., van der Lippe & Lippényi, 2020a). For instance, “high-intensity” telework (defined as working from home for more than 2.5 days per week) is negatively related to team-related performance, while “low-intensity” telework is not (see Beauregard et al., 2019).

Using a JD-R perspective, Sardeshmukh et al. (2012) found that telework was negatively related to both exhaustion and job engagement and that job demands and resources mediated these relationships. Overall, scientific literature has identified four different factors that are of utmost importance when implementing teleworking practices for employees: first, the adaptability of the work-role for teleworking, which is the most obvious parameter; second, the presence of a specific place at home to work from, with access to technology and minimal interruptions, which Baruch (2001) called the “home/work interface”; and third, support of the organization and management for employees to telework; and finally, the individual’s characteristics and need for telework (see Baruch, 2001; Saba & Cachat-Rosset, 2020).

#### Literature on forced teleworking in the context of the COVID-19 crisis

There are very few existing studies on the impact of COVID-19 on working conditions, although there are several publications based on descriptive data. A quick search, using the Web of Science tool, with keywords such as “telework* AND COVID-19” or “homebased working* AND COVID-19” yielded 54 references. We restricted the research to the year 2020 and 2021 because we are focusing on the current pandemic; the ongoing COVID-19 scenario is unique in the contemporary world of work and is definitely not comparable to other home office experiences, notably owing to the lockdown and the closure of shops and schools. Thirteen of these studies specifically adopted a management, business, or sociology lens, whereas other studies approached the problem from a gender perspective. Only a couple of these studies that evaluated the effect of forced teleworking on work characteristics, work climate, and employees’ performance, engagement, and health were relevant to our research questions. For instance, Bolisani et al. (2020) highlighted the importance of and difficulty in maintaining social relationships and contacts in this specific working context. Nevertheless, they concluded that it was neither possible to derive fully positive or negative conclusions about work from home nor feasible to get clarity about the effectiveness of these new working modalities.

On the same subject, a study (Mohring et al., 2020) assessed whether the lockdown policies (remote work, short-time work, and closure of schools and childcare) exerted an effect on family and work satisfaction among the population. Relying on individual panel data collected before and during the lockdown, they demonstrated a general decrease in family satisfaction and an overall decline in work satisfaction as well.

Carillo et al. (2021) analyzed a sample of 1,574 teleworkers in France during the lockdown. They found that crisis-specific factors influenced the adjustment of teleworkers; lack of contact and informal relationships with colleagues, as well as a lack of feedback from the manager and the organization at large, were identified as major obstacles to telework adjustment. They also underlined the importance of physical conditions (having a functional work space at home) and of being able to concentrate without disruption in the case of teleworkers. Saba and Cachat-Rosset (2020) investigated a Canadian population that teleworked during the Canadian lockdown between April 4, 2020, and July 30, 2020. Their sample (*N* = 6,750) reported an increase in workload and modification of tasks. That is, even as respondents suffered on account of being socially isolated from their colleagues and their organization, they felt more productive and were able to find a work–life balance. On the contrary, in a study that was undertaken in Germany, Abdel Hadi et al. (2021) found out that daily job demands and home demands during telework were positively related to emotional exhaustion. Finally, a study undertaken in China by Wang et al. (2021) identified four remote work challenges for employees: work–home interference, ineffective communication, procrastination, and loneliness. They underlined four virtual work characteristics that served as a buffer against these challenges: social support, job autonomy, monitoring, and workload.

The aforementioned studies emphasize the importance of job resources such as autonomy, a positive climate of work–life balance in the organization, positive relationships with colleagues, support from the organization, and flexibility. It is interesting to note that in the case of the effects of teleworking on employee outcomes, similar antecedents have been reported both in the context of forced teleworking during a crisis and in the absence of such a crisis.

#### Literature on NWW

Given that telework is a type of flexible work arrangement, it is interesting to see what the literature on the NWW has shown so far. NWW is a human resource management approach, which was introduced in many organizations globally and facilitated by the development of new information technologies (e.g., mobile devices and internet facilities; Blok et al., 2011; de Leede & Nijland, 2017; Gerards et al., 2018). NWW constitutes forms of work that allow workers to choose when and where they work and that involve the use of ICT to easily access colleagues and supervisors (Nijp et al., 2016; ten Brummelhuis et al., 2012).

Previous studies on NWW have pointed out that it may positively impact employees’ well-being (Gerards et al., 2018; Peters et al., 2014; van der Voordt, 2003) as well as employees’ performance (ten Brummelhuis et al., 2012). Other studies have highlighted that NWW does not exert any positive or negative effect on employees’ well-being or performance (Blok et al., 2012; Nijp et al., 2016; Van Steenbergen et al., 2017). Kingma (2019) pointed out that employees experienced great difficulty in coping with NWW and highlighted the negative impact of these practices on employees’ health, work engagement, and social cohesion. Nijp et al. (2016) showed that NWW may prompt employees to invest additional hours at work, but they did not identify any particular impacts of NWW on work–life balance, performance, or employee health. However, ten Brummelhuis et al. (2012) found that daily use of NWW was positively related to daily work engagement and negatively related to daily exhaustion due to increased effective and efficient communication.

### Research Hypotheses

Based on existing literature, two dimensions related to NWW were taken into account in this study: the actors’ ability to freely decide their work schedule and place of work (organizational freedom) and their ability to quickly reach colleagues, team members, or managers (easy access to colleagues and managers). Indeed, the COVID-19 crisis has had a direct impact on these two dimensions.

Second, according to previous research (Bolisani et al., 2020; Carillo et al., 2021; Saba & Cachat-Rosset, 2020), in the context of teleworking, face-to-face interactions with coworkers and managers diminish, which can affect employees’ perceptions of relatedness to their team and the organization. At the same time, according to NWW literature (Gerards et al., 2018; Peters et al., 2014; van der Voordt, 2003), telework offers the advantage of reducing the stress associated with commuting and, hence, may reduce the employee’s perception of the mental demands of their job. These two NWW dimensions (organizational freedom and easy access to colleagues and managers) can act as resources for public servants and positively impact their engagement and perceived performance while negatively impacting their level of exhaustion in the context of forced telework. Accordingly, we formulated the following hypotheses:

**Hypothesis 1:** The positive association between organizational freedom with employee engagement as well as with perceived performance is stronger during than before the forced teleworking period, while the negative association between organizational freedom with employee exhaustion is stronger during than before the forced teleworking period.

Furthermore, the literature shows that the use of NWW practices, in particular the ability to easily keep in touch with colleagues and the hierarchy, is considered to be a resource for sustaining engagement and reducing employee exhaustion (see ten Brummelhuis et al., 2012). This result leads us to two additional hypotheses:

**Hypothesis 2:** The positive association between easy access to colleagues and managers with engagement and perceived performance is stronger during than before forced telework, whereas the negative association between easy access to colleagues and managers with exhaustion is stronger during than before forced telework.

According to the literature review, with respect to teleworking and forced teleworking, several job-related factors seem to be of great importance when considering work outcomes (engagement, exhaustion, and perceived performance). More specifically, autonomy in the workplace (i.e., having a job that allows for a great deal of independence in carrying out tasks as well as for the ability to use one’s own judgment) is an important resource identified in the literature (Wang et al., 2021), particularly with respect to understanding the link between teleworking and organizational commitment or well-being at work. The Job-Characteristics Model (Hackman & Oldham, 1976; Xanthopoulou et al., 2009) stresses that the ability to work on a variety of tasks and, therefore, to use a variety of knowledge and skills may be considered as important job resources. Given that the importance of these two job-related variables, namely, autonomy and diversity in skills and tasks, is supported by previous research, we developed some more hypotheses:

**Hypothesis 3:** The positive association between autonomy in the workplace with employee engagement and perceived performance is stronger during than before forced telework period, whereas the negative association between autonomy in the workplace with employee exhaustion is stronger during than before forced telework period.
**Hypothesis 4:** The positive association between a variety of tasks and skills with employee engagement and perceived performance is stronger during than before forced telework period, whereas the negative association between a variety of tasks and skills with employee exhaustion is stronger during than before forced telework period.

As indicated by the literature review, social isolation can be one of the consequences of teleworking (Saba & Cachat-Rosset, 2020). The relational aspect of work—in particular, the opportunity to maintain contact with colleagues and to benefit from their support—is an important resource that helps to limit the deleterious effects of social isolation (see, for instance, Park et al., 2021). Therefore, we formulate one more hypothesis:

**Hypothesis 5:** The positive association between support from colleagues with employee engagement and perceived performance is stronger during than before forced telework period, whereas the negative association between support from colleagues with employee exhaustion is stronger during than before forced telework period.

Finally, the literature review revealed that a work climate favorable to telework is important for generating positive feelings toward this specific work modality. However, while some studies have highlighted the difficulty that employees face in reconciling their personal and professional lives during forced telework, other studies have shown that forced telework led to a better work–life balance (Mohring et al., 2020; Wang et al., 2021). In any case, employees’ perception of the degree of openness of their direct supervisor, or of their organization as a whole, to the work–life balance issue is a dimension of the organizational climate that can clearly impact employees’ engagement, exhaustion, and perceived performance. Thus, a favorable work–life balance climate positively affects work engagement and performance, even among street-level bureaucrats (Destler, 2017). Accordingly, we formulated the last hypothesis:

**Hypothesis 6:** The association between positive work–life balance climate with employee engagement and perceived performance is stronger during than before forced telework period, whereas the negative association between positive work–life balance climate with employee exhaustion is stronger during than before forced telework period.

### Research Model

**Figure 1.**
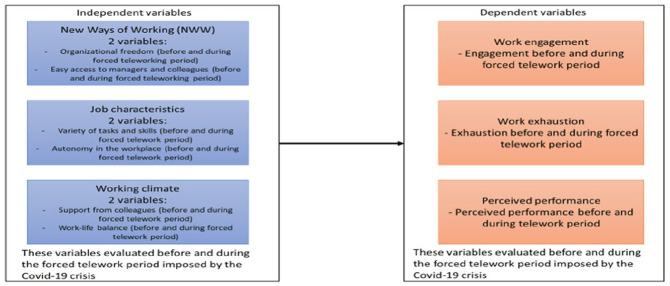
Research Model.

Based on our review of the literature with respect to the JD-R Model, as well as the facilitating and detrimental factors for teleworking, NWW, and forced teleworking during COVID-19, we identified several dependent (employee engagement, exhaustion, and perceived performance) and independent variables: two variables related to forced telework characteristics (organizational freedom and easy access to colleagues and managers), two variables related to job characteristics (autonomy in the workplace and variety of tasks and skills), one variable related to the perception of support in the workplace (support from colleagues), and finally, one variable related to the perceived organizational climate (work–life balance). Figure 1 depicts the research model developed for the present study, comprising of all these variables.

## Method

### Sample and Procedure

To investigate the relationships between the different variables included in our research model, we adopted a quantitative methodology. Keeping in mind the pandemic and telework conditions, the survey method was deemed to be most suitable for collecting data from participants. Data were collected from a single Swiss Cantonal administration (name withheld to ensure anonymity and confidentiality) located in the French-speaking part of the country. This is one of the most important Cantons in Switzerland in terms of population size as well as in economic terms. A large sample was recruited from almost all the departments of this organization. To optimize the response rate in our online survey, we contacted the HR Department of the Canton, whereupon its executive members gave their official approval of this study. The questionnaire was developed in partnership with the leaders of the HR Department. After the test phase, an internet link to the questionnaire was sent to the HR Department, which invited the employees to fill the electronic questionnaire within 3 weeks (May 25, 2020–June 12, 2020). A reminder was sent after 1.5 weeks, prompting all the employees to complete their questionnaires. Furthermore, to ensure complete privacy, answers were directly saved on a server belonging to our university. Thus, employees did not have access to the data, and the respondents were completely and transparently informed about the research procedure. This announcement of procedures served the following two purposes: to increase the participation rate and to function as a baseline requirement to reduce common method bias (Podsakoff et al., 2003). A single questionnaire was administered to the participants who were asked to answer the same question from two different perspectives: before the period of forced telework and during the period of forced telework.

Out of 3,223 public employees, 1,373 completed our questionnaire (return rate of 42.6%), which is quite substantial for this type of research. Other studies conducted in Switzerland in recent years with large samples have had similar response rates. For instance, research by Petrovsky and Ritz (2014) reported a return rate of 56.23% for a sample of 26,544 respondents, whereas Giauque et al. (2011) reported a return rate of 38.1% for a sample of 9,852 respondents. However, as we were denied access to the HR data for all the departments in the canton administration owing to data confidentiality concerns, we could not make a clear judgment about the representativeness of our sample; this represents a methodological limitation of our study. Nevertheless, having a large sample size provides some assurance of the robustness of our findings.

Women comprised 70.5% of our sample and 51.1% of all respondents reported having dependent children at home. In addition, 19.1% of the participants were supervisors, 30.2% of the participants were between the ages of 19 and 40 years, 64.2% were between 40 and 60 years, and 5.6% were aged 60 years or older. Furthermore, the level of education in our sample was high: 29.9% had been in a vocational track (elementary schools to professional baccalaureate), whereas 68.3% had an academic background (college degree to university diploma). Regarding organizational tenure, 30.2% of the participants had been with their current organization for less than 1 or up to 5 years, whereas 59.8% had been with the organization for 5 to 10 years.

### Measures

Most of the items^1^ was measured using 5-point Likert-type scales, with the endpoints *strongly disagree* (1) and *strongly agree* (5). The instruments relied on self-reports. It should be recalled here that the respondents became acquainted with the variables by putting themselves in a situation before and during the forced telework period. They answered the same questions (same variables) for the two time periods—before and during the Covid-19 crisis. Hence, the explanations of the measures below concern responses for the periods before and during the forced telework period—items before and during the forced telework period were placed in two separate parts of the questionnaire, to avoid bias and confusion in responses.

#### Independent variables (both before and during COVID)

##### NWW

To measure this variable, we relied on items already tested in previous research on NWW. Based on factorial analysis of these items, we were able to isolate two variables related to work arrangements. The first relates to the actors’ ability to decide freely their work schedule and place of work. We will call it organizational freedom (3 items). The second isolated variable related to work arrangements is linked to the ability of the actors to quickly reach colleagues, team members, or their managers. This variable, created based on 3 items, is called easy access to colleagues and managers.

##### Job characteristics

We created two variables related to the dimension of job characteristics. These two variables are inspired by the job characteristics model (Hackman & Oldham, 1975, 1976), and the items used to construct these variables are extracted from an already tested measurement scale (Kim, 2016). The first variable measures the diversity of the tasks and skills involved in the job. We will call it a variety of tasks and skills. The second variable is related to the respondents’ autonomy in doing their job as well as the possibility of taking initiatives. We will call it autonomy in the workplace.

##### Working climate

This variable focuses on the actors’ perception of the work climate in which they work. Based on a factorial analysis, we were able to develop two variables related to this dimension. The first relates to the actors’ perception of their relationships with colleagues. This measure is taken from a validated scale and includes 3 items (Euromed, 2015). We will call it support from colleagues. The second variable is related to the perception of the actors regarding the presence or absence of a favorable organizational climate in terms of work–life balance. Two items are drawn from a measure already used in research as well (Thompson et al., 1999). We refer to this variable as work–life balance.

#### Dependent variables (both before and during Covid)

##### Work engagement

The 5 items comprising this variable were selected from previous studies (Seppälä et al., 2008).

##### Work exhaustion

This variable comprises 3 items extracted from a measure that has already been tested and validated (Kim, 2005; Schaufeli et al., 1995).

##### Self-rated performance

The third dependent variable is a measure of performance, which is considered an in-role performance measure (Palvalin et al., 2015). The 3 items comprising this variable were also taken from a previous study. We call this variable perceived performance.

##### Control variables

The control variables are as follows: gender (0 = *men*; 1 = *women*); children, which is related to the fact that some respondents have children (0 = *no*; 1 = *yes*); the level of education (0 = *other* to 6 = *University degree*); organizational tenure (from 1 = *less than 1 year* to 5 = *more than 10 years*); age (in number of years); and managing or having to manage a team (0 = *no*; 1 = *yes*).

### Statistical Analysis

Prior to assessing the reliability of our different variables, two supplementary indicators were used to test the condition of the dataset, that is, to ensure that the assumptions of normality were upheld and determine the presence of multicollinearity (notably, the assumption of normality pertains to residuals, not the survey data itself). The tolerance and variance inflation factor scores of our data also fell within the acceptable range for all the variables. Based on this evidence, we conclude that the dataset was in a good condition.

The first phase of our statistical tests focused on applying tests of means (*t* test procedures, using the Stata 16 software) on the variables to determine whether our respondents responded significantly differently to the same items when they related to the situation before and during the forced telework period.

In the second step, we wanted to better understand the effects of the independent and control variables on the dependent variables. Hence, we conducted three regression analyses (ordinary least square regressions using Stata 16). This was done to identify which variables correlated most closely with the three dependent variables, before and during the crisis. Multicollinearity and heteroskedasticity tests were performed on each regression. We did not detect multicollinearity problems; using Stata 16, we corrected the heteroskedasticity problems.

## Results

### Two-Tailed *t* Tests Results

We will begin by presenting the results of our means tests in relation to our respondents’ answers regarding their perceptions before and during the forced telework period. The results are summarized in Table 1.

**Table 1.** Two-Tailed Tests Summary.

Two-tailed test (without any direction):	Interpretation of the results: mean answers to the different variables before and during the forced telework period
Organizational freedom before <-> Organizational freedom during	Two-tailed test, *t*(1,368)= –38.12, *p* < .0000, statistically significant.
Easy access to colleagues and managers before <-> Easy access to colleagues and managers during	Two-tailed test, *t*(1,367)= 5.93, *p* < .0000, statistically significant
Variety of tasks and skills before <-> Variety of tasks and skills during	Two-tailed test, *t*(1,366)= 4.85, *p* < .0000, statistically significant
Autonomy in the workplace before <-> Autonomy in the workplace during	Two-tailed test, *t*(1,365)= –7.60, *p* < .0000, statistically significant
Support from colleagues before <-> Support from colleagues during	Two-tailed test, *t*(1363)= 4.38, *p* < .0000, statistically significant
Work–life balance before <-> Work–life balance during	Two-tailed test, *t*(1,363)= –6.39, *p* < .0000, statistically significant
Engagement before <-> Engagement during	Two-tailed test, *t*(1,366)= 1.36, *p* < .1742, not statistically significant
Exhaustion before <-> Exhaustion during	Two-tailed test, *t*(1,366)= 8.31, *p* < .0000, statistically significant
Perceived performance before <-> Perceived performance during	Two-tailed test, *t*(1,351)= 7.84, *p* < .0000, statistically significant

In Table 1, based on the averages of the responses, respondents feel that their freedom to organize work, in terms of schedule and location, was higher during than before the forced telework period. On average, respondents feel that it was easier to contact colleagues and supervisors before than during the forced telework period. They also felt that the period before the forced telework period allowed them to engage in more diverse activities and, therefore, to apply a wider range of skills. However, the forced telework situation allowed them to enjoy a higher autonomy, as their independence and personal initiatives increased during this period compared with the prevailing situation. Cooperation between colleagues suffered during the forced telework period; on average, several employees felt that the forced telework situation was less favorable to social relationships with colleagues than the situation before the forced telework period. On average, they also believed that the climate for the work–life balance was more favorable during the forced telework period than before it.

In terms of engagement, our statistical analyses do not show differences in average responses between the situation before the forced telework period and during the crisis. However, the means in relation to exhaustion show that our respondents perceive the period of forced telework as being more favorable to their health. Finally, our respondents perceived the period before the forced telework to be more favorable to their individual performance than that during the forced telework period.

### Results of the Ordinary Least Squares Regression Analyses

We identify the antecedents of the three dependent variables (engagement, exhaustion, and perceived performance).

#### Antecedents of engagement before the forced telework period

Our first ordinary least squares (OLS) regression analysis (Table 2) explains about 33% of the variance of respondents’ engagement, which is significant. We observed high engagement in the case of women, younger people, those with the least organizational tenure, and those with lower levels of education. Respondents with hierarchical responsibilities were more likely to report high levels of engagement with their work. At an organizational level, variables related to job characteristics and organizational climate were found to be important explanatory factors for respondents’ job engagement. In fact, the job characteristics such as the ability to conduct a variety of activities, the use of a variety of skills, greater freedom to organize work, and the opportunity to take personal initiatives are considered resources for the actors. These resources increase the work engagement of the actors. We also observe the significance of aspects related to the work climate. A good working atmosphere with colleagues, as well as an organizational climate conducive to work–life balance, is an important resource for the respondents. It is important to point out that, before the forced telework period, the two aforementioned variables related to NWW were not statistically significantly related to engagement.

**Table 2.** OLS Regression Regarding Engagement Before and During Lockdown.

Engagement before lockdown	Coef.	*SE*	*t* values	Engagement during lockdown	Coef.	*SE*	*t* values
Gender	.100	.037	2.69**	Gender	.050	.038	1.30
Children at home	–.029	.033	–.086	Children at home	.004	.034	.12
Age	.006	.002	3.30**	Age	.004	.002	2.12*
Level of education	–.020	.008	–2.42*	Level of education	–.031	.008	–3.62***
Tenure	–.052	.014	–3.64***	Tenure	–.020	.014	–1.41
Manager (yes or no)	.112	.043	2.57*	Manager (yes or no)	.007	.044	.16
Organizational freedom	–.021	.016	–1.32	Organizational freedom	–.009	.018	–.50
Easy access to colleagues and managers	.020	.024	0.84	Easy access to colleagues and managers	.156	.026	5.97***
Variety of tasks and skills	.145	.026	5.41***	Variety of tasks and skills	.174	.028	6.07***
Autonomy in the workplace	.174	.027	6.35***	Autonomy in the workplace	.307	.032	9.50***
Support from colleagues	.260	.028	9.18***	Support from colleagues	.174	.030	5.70***
Work–life balance	.223	.024	9.23***	Work–life balance	.181	.027	6.63***
Number of observations:	1,243			Number of observations:	1,245		
*F* statistic	45.20***			*F* statistic	73.25***		
*R* ^2^	.335			*R* ^2^	.43		
Root Mean Squared Error (MSE)	.579			Root MSE	.595		

*Note.* OLS = ordinary least squares.

* *p* < .05. **p < .01. ***p < .001.

#### Antecedents of engagement during the forced telework period

If we now turn to our regression (Table 2) in relation to the engagement of our respondents during the forced telework period (43% of the variance of engagement explained by our variables), we get somewhat similar results, except for the sociodemographic aspects. Older respondents and those reporting a low level of education are more likely to declare themselves engaged during the forced telework period. However, the same organizational variables identified in the previous regression had a statistically positive impact on our respondents’ work engagement. The only difference is that, during the forced telework period, the ability of the actors to collaborate with colleagues, supervisors, and team members plays a crucial role in increasing engagement. Hence, this aspect determines the engagement of respondents during forced teleworking.

#### Antecedents of exhaustion before the forced telework period

The variables included in our regression (see Table 3) explain 13% of our respondents’ exhaustion. Surprisingly, respondents without children at home reported higher levels of exhaustion than those with children. It is possible that this exhaustion is related to the number of people, not children, living in the same household. However, in the present study, as we do not have this information, it creates a limitation for explaining this finding. Respondents with a shorter tenure also reported higher levels of exhaustion. Respondents, who felt they had a variety of tasks to perform, and therefore, a variety of skills to apply in their work activities, also reported higher levels of exhaustion. However, independence, autonomy at work, and organizational climate aspects (good relationships with colleagues and a climate favorable to work–life balance) are factors that protect the respondents from exhaustion. Once again, the two variables related to NWW were not related to exhaustion before the forced telework period.

**Table 3.** OLS Regression Regarding Exhaustion Before and During Lockdown.

Exhaustion before lockdown	Coef.	*SE*	*t* values	Exhaustion during lockdown	Coef.	*SE*	*t* values
Gender	–.087	.063	–1.38	Gender	–.125	.063	–1.96*
Children at home	–.141	.056	–2.50*	Children at home	.048	.056	.86
Age	–.006	.003	–1.95	Age	–.001	.003	–.55
Level of education	.000	.014	.04	Level of Education	.010	.013	.78
Tenure	.085	.024	3.51***	Tenure	.025	.023	1.05
Manager (yes or no)	–.096	.075	–1.28	Manager (yes or no)	.144	.077	1.86
Organizational freedom	.016	.027	.61	Organizational freedom	–.117	.029	–4.03***
Easy access to colleagues and managers	–.070	.038	–1.83	Easy access to colleagues and managers	–.102	.041	–2.47*
Variety of tasks and skills	.141	.042	3.31**	Variety of tasks and skills	.167	.041	4.05***
Autonomy in the workplace	–.163	.038	–4.28***	Autonomy in the workplace	–.200	.048	–4.13***
Support from colleagues	–.166	.045	–3.69***	Support from colleagues	–.044	.047	–.93
Work–life balance	–.257	.037	–6.90***	Work–life balance	–.151	.037	–3.99***
Number of observations:	1,243			Number of observations	1,245		
*F* statistic	15.73***			*F* Statistic	12.67***		
*R* ^2^	.13			*R* ^2^	.13		
Root Mean Squared Error	.982			Root MSE	.969		

*Note.* OLS = ordinary least squares.

* *p* < .05. **p < .01. ***p < .001.

#### Antecedents of exhaustion during the forced telework period

Variables included in this regression also explain 13% (Table 3) of exhaustion during the forced telework period. Our male respondents were more likely (just statistically significant) to report higher levels of exhaustion. Two factors related to the NWW were negatively related to exhaustion: (a) the ability to freely determine the work schedule and location and (b) the ability to collaborate with colleagues, supervisors, and team members. High work autonomy and the freedom to use personal initiatives were negatively related to exhaustion. The other one (variety of tasks and skills) was positively related to exhaustion during forced telework period (a result similar to exhaustion before the forced telework period). Finally, a climate favorable to work–life balance was also negatively related to exhaustion during the forced telework period.

#### Antecedents of perceived performance before the forced telework period

Our regression analysis reveals that our variables explain 14% of the perceived performance during the forced telework period (see Table 4). Respondents with lower levels of education reported higher levels of perceived performance. Easy access to colleagues and managers, a variety of tasks and skills, autonomy in the workplace, and support from colleagues were all statistically significantly related to higher levels of perceived performance.

**Table 4.** OLS Regression Regarding Perceived Performance Before Lockdown and During Lockdown.

Perceived performance before lockdown	Coef.	*SE*	*t* values	Perceived performance during lockdown	Coef.	*SE*	*t* values
Gender	.031	.037	.82	Gender	.040	.044	.90
Children at home	–.058	.033	–1.78	Children at home	–.017	.040	–.44
Age	–.000	.001	–.13	Age	–.000	.002	–.34
Level of education	–.027	.008	–3.31**	Level of education	–.038	.010	–3.81***
Tenure	.021	.013	1.60	Tenure	.040	.016	2.41*
Manager (yes or no)	–.030	.040	–.75	Manager (yes or no)	–.008	.047	–.19
Organizational freedom	.004	.015	.27	Organizational freedom	.089	.022	4.03***
Easy access to colleagues and managers	.128	.025	5.03***	Easy access to colleagues and managers	.257	.032	7.95***
Variety of tasks and skills	.095	.031	3.02**	Variety of tasks and skills	.135	.035	3.85***
Autonomy in the workplace	.099	.028	3.50***	Autonomy in the workplace	.321	.040	8.02***
Support from colleagues	.122	.029	4.18***	Support from colleagues	.079	.037	2.13*
Work–life balance	.038	.201	11.44***	Work–life balance	–.019	.031	–.16
Number of observations:	1,230			Number of observations	1,245		
*F* statistic	13.57***			*F*-statistic	46.33***		
*R* ^2^	.14			*R* ^2^	.33		
Root Mean Squared Error	.572			Root MSE	.702		

*Note.* OLS = ordinary least squares.

* *p* < .05. **p < .01. ***p < .001.

#### Antecedents of perceived performance during the forced telework period

Our last regression analysis shows that all the included variables explain 33% of the variance of the perceived performance during the forced telework period, which is much more than before the forced telework (see Table 4). Respondents who were less educated and had a longer tenure were more likely to report higher levels of perceived performance. Furthermore, five of six organizational factors were positively and statistically significantly related to perceived performance during the forced telework period—organizational freedom, easy access to colleagues and managers, variety of tasks and skills, and support from colleagues.

Table 5 summarizes the results of our regressions and identifies resources or demands according to our different dependent variables. It allows us to test our six hypotheses.

**Table 5.** Synthesis of the Variables Identified as Job Resources or Job Demands According to Our Dependent Variables.

Outcome variables	Identified job resources before lockdown	Identified job resources during lockdown	Identified job demands before lockdown	Identified job demands during lockdown
Engagement	Variety of tasks and skills (+) Autonomy in the workplace (+) Support from colleagues (+) Work–life balance (+)	Easy access to managers and colleagues (+) Variety of tasks and skills (+) Autonomy in the workplace (+) Support from colleagues (+) Work–life balance (+)	––	––
Exhaustion	Autonomy in the workplace (−) Support from colleagues (−) Work–life balance (−)	Organizational freedom (−) Easy access to managers and colleagues (−) Autonomy in the workplace (−) Work–life balance (−)	Variety of tasks and skills (+)	Variety of tasks and skills (+)
Perceived performance	Easy access to managers and colleagues (+) Variety of tasks and skills (+) Autonomy in the workplace (+) Support from colleagues (+)	Organizational freedom (+) Easy access to managers and colleagues (+) Variety of tasks and skills (+) Support from colleagues (+)	––	––

(+) = positively and statistically significantly related to the dependent variable (*p* < .05).

(−) = negatively and statistically significantly related to the dependent variable (*p* < .05).

Based on this summary table of our main results, we can highlight that the majority of our hypotheses are not supported by our research data. H1 is partially supported by our data. Organizational freedom is indeed a factor that positively impacts perceived performance but also negatively impacts exhaustion during the telework period, but this factor is not associated with the engagement of our respondents. H2 is only partially supported as well. Easy access to colleagues and managers is more important during forced telework period than before with regard to engagement and exhaustion. But this variable is also a resource to support perceived performance before and during forced telework. H3 is not verified in this research. Autonomy in the workplace is not a more important factor during the forced telework period than before. This factor has a positive effect on engagement, exhaustion and perceived performance, but the impact is no greater during than before the forced telework period. H4 is clearly not supported. On the contrary, this factor (variety of tasks and skills) could be identified as a factor of work arduousness as it contributes to increased exhaustion both before and during the forced telework period. H5 does not hold true in this research either. Support from colleagues is a dimension that decreases sharply during the forced telework period, and, therefore, this factor cannot be a resource for our respondents to deal with exhaustion. Finally, H6 is only partially supported by our data. A favorable work–life balance climate is not, however, associated with perceived performance either before or during the forced telework period.

## Discussion

In general, our data reveal moderate impacts of the forced telework situation on employees’ perception of their working conditions. We report a positive relationship between forced telework and job autonomy as well as between forced telework and work–life balance. On average, our respondents felt that they had more freedom to organize their work, which is one of the dimensions of NWW (organizational freedom) and that they had more opportunities to use personal initiative and judgment during the forced telework period. They also reported that the forced teleworking period was conducive to the development of a climate that allowed for a better work–life balance. This particular result calls into question certain studies, which have shown that forced telework has a negative effect on work–life balance (Mohring et al., 2020).

At the same time, other results may be more worrisome for both organizations and employees. On average, our respondents believed that the forced telework situation reduced their opportunities to collaborate with colleagues, team members, or supervisors. Thus, forced teleworking had a negative impact on the second dimension of NWW, namely, easy access to colleagues as well as on the dimension related to collaboration within the work climate. The respondents also experienced a decline in diversity in their tasks and their work skills.

Forced telework does not appear to have influenced our respondents’ level of engagement, with the averages of their responses being almost identical before or during the forced telework period. This sends a positive message to the organizations that employees can exhibit high motivation and work engagement even without having a physical connection with the organization. The other good news is somewhat counterintuitive to previous research results (Kingma, 2019), as the level of exhaustion dropped sharply during the telework period. Teleworking lowered the average level of perceived job strain of our respondents. One explanation may lie in the fact that new work arrangements have enhanced the work–life balance by facilitating a better integration of constraints related to private activities. Another potential explanation comes from Abdel Hadi et al.’s (2021) study, which demonstrates the importance of leisure crafting to reduce exhaustion during forced teleworking. Furthermore, these results are consistent with those of previous empirical studies that have indicated that NWW may be favorable for employees’ health (Diab-Bahman & Al-Enzi, 2020; Peters et al., 2014; van der Voordt, 2003).

However, on average, our respondents felt that their performance dipped and was negatively impacted during the forced telework period. It is necessary to consider employees’ perceptions of their performance, as these perceptions are relevant to measuring workplace outcomes (Hewett et al., 2018) and maybe as important as the employees’ actual performance. This result can possibly be explained by the decline in feedback during forced teleworking; a decline in the quality of relationships with coworkers and the inability to collaborate with colleagues and supervisors hinders the employee feedback and work support systems. Consequently, under such conditions, it becomes functionally more difficult to perform. However, a Canadian study by Saba and Cachat-Rosset (2020) found that approximately half of their sample of 6,750 respondents felt they were more productive during the period of forced teleworking. These contradictory results call for the need to gather more evidence on this matter.

Our OLS regression analyses showed that NWW dimensions and work–life balance are positively related to work engagement during the forced telework period. We identified several variables that constitute resources for the employees. Some of these resources can contribute toward lessening the negative impacts of forced telework. For example, employees must have certain job characteristics—the ability to perform a diversified job, the use of a variety of skills, freedom to organize one’s own work, and the opportunity to use personal initiative and judgment. Our survey confirms that these characteristics of work, which have been extensively studied in the scientific literature (Bakker & Demerouti, 2007; Bakker et al., 2010), are important to ensure the work engagement of public employees.

Moreover, aspects of organizational climate also contribute toward employee engagement and occupational health. Our data underline that the perception of a good understanding and collaboration with colleagues is central to engagement at all times. In this case, our survey confirms the results of previous studies (Destler, 2017; Pecino et al., 2019). It should also be noted that the more the respondents perceived that their supervisors and, in general, their organization were in favor of a good work–life balance, the higher was their work engagement and the lesser was their exhaustion (Wood et al., 2020). Thus, a positive work–life balance climate is an important resource for employees, both during normal and forced telework periods. These results are consistent with the ones obtained by other studies focusing on the lockdown period (Bolisani et al., 2020; Saba & Cachat-Rosset, 2020; Wang et al., 2021).

If the aforementioned resources limit work exhaustion, then the variety of tasks and skills can act as a double-edged variable. We found a positive and significant relationship between this specific job-related variable and the other two dependent variables—engagement and exhaustion. In relation to this independent variable, there is a clear trade-off. It is a resource if it exerts a positive effect on engagement. However, it also represents a job demand because it fosters exhaustion. Indeed, other studies (Grant et al., 2007; Van De Voorde et al., 2012) have shown that certain aspects of work can both generate more engagement or satisfaction and have a negative impact on workers’ health. It can also contribute toward exhaustion, especially in times of crisis and forced teleworking. It should also be noted that the relational climate with colleagues proves to be a resource in normal times, but logically loses its protective value during a forced telework period. As teleworking is not favorable to social relations, this result seems logical.

Finally, it is also useful to mention the important role played by the freedom to determine one’s own work schedule and workplace and the easy access to colleagues and supervisors during the forced telework period. Related to NWW, these variables negatively impact exhaustion, positively impact individual engagement, and contribute toward shaping a more favorable perception of self-performance. In our study, these variables are important in a context of a forced teleworking situation. Further research is needed to find whether those two NWW variables could be useful also in a “normal” teleworking context.

### Limitations

Nevertheless, this study has several limitations. First, even if the variables used in this study capture a non-negligible proportion of the variance of our dependent variables, they are very likely to omit other important explanatory factors. For instance, working from home or teleworking requires technological competencies and skills as well as good IT infrastructures and materials (van der Lippe & Lippényi, 2020b). These specific facets of teleworking need further research. Individual variables may also have to be complemented. Single parents with children (forced to contribute to home-schooling) or those with dependent relatives may not find teleworking an effective work option. In our research, we included some sociodemographic variables, but individual conditions have to be better investigated. It may well be possible that other individual characteristics like personality traits can influence employees’ responses. In addition, differences may emerge depending on whether people work full-time or part-time. It is also very likely that other variables may interact with the variables we have included in our own research. For example, the organizational culture, the leadership style, or even the level of trust between employees and management may interact with variables integrated in our analysis. Thus, it would be useful to better investigate, in the future, the possible moderating or even mediating effects between these interaction variables and the variables proposed in our survey. Finally, the type and nature of activities performed by employees have not been investigated. To better understand the relationships between the selected variables, it would be crucial to include the categories of occupational work.

Furthermore, this study has several methodological limitations. First, as our data are cross-sectional in nature, we cannot determine the causal relationships between our variables. Nevertheless, if we follow DeHart-Davis et al.’s (2015) argument, theoretical reasoning is of great importance and certainly provides guidance when dealing with causal relationships. According to theoretical perspectives reviewed previously, our assumption is that modalities of work (NWW), job characteristics, and working climate precede work engagement, work exhaustion, and perceived performance. Our results are consistent with the theoretical argument, although future research must be developed to challenge our results.

Another important methodological issue is related to the one-sided methodology (i.e., a self-report survey to collect predictor and outcome variables) adopted in this study, which can result in common method biases (Podsakoff et al., 2003). This strategy may inflate the reported effect sizes. However, we tried to minimize this problem through the conditions of the survey. Another drawback related to our survey is the fact that respondents have to answer questions related to two different periods: one pertaining to before the forced telework period and another pertaining to the forced telework period. This strategy of administering only one questionnaire to collect points of view concerning two different temporalities is not immune to criticism and is probably not perfect for avoiding statistical or representational biases. Thus, while the adopted strategy was not perfect, it was the only feasible one at the time our survey was launched.

Finally, our sample comprises employees working in a large Swiss cantonal public administration. It would be interesting to conduct the same type of survey in private organizations, nongovernmental organizations (NGOs), or international organizations to compare the results and identify any differences. These methodological limitations may lead to new research perspectives.

## Conclusion and Recommendations

To the best of our knowledge, this research is the first to investigate the impacts of NWW, job resources, and work climate during the period of forced teleworking induced by the Covid-19 crisis. Our results showed the differences in certain job resources, dimensions of NWW, and the work climate, as perceived by our respondents during the forced telework period and before it. In line with the previous literature on NWW and particularly with the empirical studies undertaken during the forced teleworking period, the evidence from the present study underlines the importance of NWW (the freedom to decide the place and time to work), job resources (autonomy and variety of tasks and skills), and the work climate (positive collaboration with colleagues and favorable work-life climate) for enhancing work engagement and performance and for reducing exhaustion among public sector employees. Second, this study shed some light on the importance of positive relationships at work, a climate favorable to collaboration, and a work–life balance for employees and organizations.

### Practical Recommendations

Based on previous results, we can propose recommendations for managers and HR specialists in public organizations. In normal times, it seems necessary to allow employees to benefit from autonomy and independence in their work and equip them to carry out diverse tasks requiring the use of a variety of skills. It is also very important to develop a climate conducive to social relations and consider the importance of a better work–life balance. In times of forced telecommuting, the ability to contact colleagues and supervisors becomes crucial to fostering employee engagement, reducing exhaustion, and promoting performance development. Similarly, during a crisis, the ability to work from anywhere and at any time functions as a crucial resource. However, the option to work on diverse tasks and use a range of skills may lead to unclear consequences.

As noted earlier, there has been a significant decline in perceived performance during the forced telework period. To counteract this feeling, organizations can promote opportunities for performance feedback. Social isolation and distance from colleagues and supervisors may diminish support to carry out the job and are probably not conducive to a favorable perception in terms of performance. However, further research is needed to better inform practical recommendations.

Finally, what can our results suggest about a co-modal or hybrid work situation that organizations are increasingly moving toward? Probably we need to review leadership practices and postures, as a first step. In terms of steering organizations, managers will simply not be able to do what they used to do. They have to adapt their expectations and behaviors to the reality of partial and sustainable telework for a large proportion of employees in the future. This means trusting, making themselves available to foster links, and to give additional feedback to employees, no longer focusing on “how” the work is done but rather on the objectives. It means adopting management by objectives. This new reality of hybrid work will also require a rethinking of the ways in which managers can create favorable organizational conditions to generate social bonds between themselves and employees but also between employees. In any case, a cultural revolution is underway in the world of work. Our organizations will have to try to create a favorable climate for reconciling private and professional life, to offer more hybridity and new ways of working; otherwise, they may not be able to successfully compete in the labor market.

## Notes

1. The questionnaire and its items, as well as the table of correlations between the study variables and the Cronbach αs, are available on request. Please contact the corresponding author.

## References

[bibr1-00910260211073154] Abdel Hadi S. Bakker A. B. Häusser J. A. (2021). The role of leisure crafting for emotional exhaustion in telework during the COVID-19 pandemic. Anxiety, Stress, and Coping, 34, 530–544. 10.1080/10615806.2021.1903447 33769142

[bibr2-00910260211073154] Bakker A. B. Demerouti E. (2007). The job demands-resources model: State of the art. Journal of Managerial Psychology, 22(3), 309–328.

[bibr3-00910260211073154] Bakker A. B. Demerouti E. (2017). Job demands–resources theory: Taking stock and looking forward. Journal of Occupational Health Psychology, 22(3), 273–285. 10.1037/ocp0000056 27732008

[bibr4-00910260211073154] Bakker A. B. Demerouti E. Sanz-Vergel A. I. (2014). Burnout and work engagement: The JD–R approach. Annual Review of Organizational Psychology and Organizational Behavior, 1(1), 389–411.

[bibr5-00910260211073154] Bakker A. B. van Veldhoven M. Xanthopoulou D. (2010). Beyond the demand-control model: Thriving on high job demands and resources. Journal of Personnel Psychology, 9(1), 3–16.

[bibr6-00910260211073154] Baruch Y. (2001). The status of research on teleworking and an agenda for future research. International Journal of Management Reviews, 3(2), 113–129.

[bibr7-00910260211073154] Beauregard T. A. Basile K. A. Canonico E. (2019). Telework: Outcomes and facilitators for employees. In Landers R. N. (Ed.), The Cambridge handbook of technology and employee behavior (pp. 511–543). Cambridge University Press.

[bibr8-00910260211073154] Beurden J. V. Voorde K. V. D. Veldhoven M. V. (2020). The employee perspective on HR practices: A systematic literature review, integration and outlook. The International Journal of Human Resource Management, 32(2), 1–35.

[bibr9-00910260211073154] Blok M. Groenesteijn L. Schelvis R. Vink P. (2012). New ways of working: Does flexibility in time and location of work change work behavior and affect business outcomes? Work, 41(1), 2605–2610. 22317114 10.3233/WOR-2012-1028-2605

[bibr10-00910260211073154] Blok M. Groenesteijn L. van den Berg C. Vink P. (2011). New ways of working: A proposed framework and literature review. In Robertson M. M. (Ed.), Ergonomics and health aspects of work with computers (Vol. 6779, pp. 3–12). Springer.

[bibr11-00910260211073154] Bolisani E. Scarso E. Ipsen C. Kirchner K. Hansen J. P. (2020). Working from home during COVID-19 pandemic: Lessons learned and issues. Management & Marketing-Challenges for the Knowledge Society, 15, 458–476.

[bibr12-00910260211073154] Borst R. T. (2018). Comparing work engagement in people-changing and people-processing service providers: A mediation model with red tape, autonomy, dimensions of PSM, and performance. Public Personnel Management, 47(3), 287–313. 30135612 10.1177/0091026018770225 PMC6088520

[bibr13-00910260211073154] Borst R. T. Kruyen P. M. Lako C. J. de Vries M. S. (2019). The attitudinal, behavioral, and performance outcomes of work engagement: A comparative meta-analysis across the public, semipublic, and private sector. Review of Public Personnel Administration, 40(4), 613–640. 10.1177/0734371X19840399

[bibr14-00910260211073154] Caillier J. G. (2012). The impact of teleworking on work motivation in a US federal government agency. The American Review of Public Administration, 42(4), 461–480.

[bibr15-00910260211073154] Carillo K. Cachat-Rosset G. Marsan J. Saba T. Klarsfeld A. (2021). Adjusting to epidemic-induced telework: Empirical insights from teleworkers in France. European Journal of Information Systems, 30(1), 69–88. 10.1080/0960085x.2020.1829512

[bibr16-00910260211073154] DeHart-Davis L. Davis R. S. Mohr Z. (2015). Green tape and job satisfaction: Can organizational rules make employees happy? Journal of Public Administration Research and Theory, 25(3), 849–876.

[bibr17-00910260211073154] de Leede J. Nijland J. (2017). Understanding teamwork behaviors in the use of new ways of working. In DeLeede J. (Ed.), New ways of working practices: Antecedents and outcomes (pp. 73–94). Emerald Group Publishing Limited.

[bibr18-00910260211073154] Demerouti E. Bakker A. B. Nachreiner F. Schaufeli W. B. (2001). The job demands-resources model of burnout. Journal of Applied Psychology, 86(3), 499–512. 11419809

[bibr19-00910260211073154] Destler K. N. (2017). A matter of trust: Street level bureaucrats, organizational climate and performance management reform. Journal of Public Administration Research and Theory, 27(3), 517–534.

[bibr20-00910260211073154] de Vries H. Tummers L. Bekkers V. (2019). The benefits of teleworking in the public sector: Reality or rhetoric? Review of Public Personnel Administration, 39(4), 570–593. 10.1177/0734371x18760124

[bibr21-00910260211073154] Diab-Bahman R. Al-Enzi A. (2020). The impact of COVID-19 pandemic on conventional work settings. International Journal of Sociology and Social Policy, 40(9/10), 909–927.

[bibr22-00910260211073154] Euromed. (2015). Sixth European Working Conditions Survey 2015. European Foundation for the Improvement of Living and Working Conditions.

[bibr23-00910260211073154] Gerards R. de Grip A. Baudewijns C. (2018). Do new ways of working increase work engagement? Personnel Review, 47(2), 517–534.

[bibr24-00910260211073154] Giauque D. Anderfuhren-Biget S. Varone F. (2013). Stress perception in public organisations: Expanding the job demands–job resources model by including public service motivation. Review of Public Personnel Administration, 33(1), 58–83.

[bibr25-00910260211073154] Giauque D. Ritz A. Varone F. Anderfuhren-Biget S. Waldner C. (2011). Putting public service motivation into context: A balance between universalism and particularism. International Review of Administrative Sciences, 77(2), 227–253. 10.1177/0020852311399232

[bibr26-00910260211073154] Golden T. D. Gajendran R. S. (2019). Unpacking the role of a telecommuter’s job in their performance: Examining job complexity, problem solving, interdependence, and social support. Journal of Business and Psychology, 34(1), 55–69. 10.1007/s10869-018-9530-4

[bibr27-00910260211073154] Grant A. M. Christianson M. K. Price R. H. (2007). Happiness, health, or relationships? Managerial practices and employee well-being tradeoffs. Academy of Management Perspectives, 21(3), 51–63.

[bibr28-00910260211073154] Hackman J. R. Oldham G. R. (1975). Development of the job diagnostic survey. Journal of Applied Psychology, 60(2), 159–170.

[bibr29-00910260211073154] Hackman J. R. Oldham G. R. (1976). Motivation through the design of work: Test of a theory. Organizational Behavior and Human Performance, 16(2), 250–279.

[bibr30-00910260211073154] Hewett R. Shantz A. Mundy J. Alfes K. (2018). Attribution theories in human resource management research: A review and research agenda. The International Journal of Human Resource Management, 29(1), 87–126.

[bibr31-00910260211073154] Karasek R. A. (1979). Job demands, job decision latitude, and mental strain: Implications for job redesign. Administrative Science Quarterly, 24(2), 285–308.

[bibr32-00910260211073154] Kim S. (2005). Factors affecting state government information technology employee turnover intentions. The American Review of Public Administration, 35(2), 137–156.

[bibr33-00910260211073154] Kim S. (2016). Job characteristics, public service motivation, and work performance in Korea. Gestion et Management Public, 5/1(3), 7–24.

[bibr34-00910260211073154] Kingma S. (2019). New ways of working (NWW): Work space and cultural change in virtualizing organizations. Culture and Organization, 25(5), 383–406.

[bibr35-00910260211073154] Kriesi H. Trechsel A. H. (2008). The politics of Switzerland: Continuity and change in a consensus democracy. Cambridge University Press.

[bibr36-00910260211073154] Mohring K. Naumann E. Reifenscheid M. Wenz A. Rettig T. Krieger U. Friedel S. Finkel M. Cornesse C. Blom A. G. (2020). The COVID-19 pandemic and subjective well-being: Longitudinal evidence on satisfaction with work and family. European Societies, 23, S601–S617. 10.1080/14616696.2020.1833066

[bibr37-00910260211073154] Nijp H. H. Beckers D. G. J. van de Voorde K. Geurts S. A. E. Kompier M. A. J. (2016). Effects of new ways of working on work hours and work location, health and job-related outcomes. Chronobiology International, 33(6), 604–618. 27223247 10.3109/07420528.2016.1167731

[bibr38-00910260211073154] Nilles J. M. Carlson J. F. R. Gray P. Hanneman G. J. (1976). The telecommunications-transportation tradeoff: Options for tomorrow. John Wiley and Sons.

[bibr39-00910260211073154] Palvalin M. Vuolle M. Jääskeläinen A. Laihonen H. Lönnqvist A. (2015). SmartWoW—constructing a tool for knowledge work performance analysis. International Journal of Productivity and Performance Management, 64(4), 479–498.

[bibr40-00910260211073154] Park S. Jeong S. Chai D. S. (2021). Remote e-Workers’ psychological well-being and career development in the era of COVID-19: Challenges, success factors, and the roles of HRD professionals. Advances in Developing Human Resources, 23(3), 222–236. 10.1177/15234223211017849

[bibr41-00910260211073154] Pecino V. Mañas M. A. Díaz-Fúnez P. A. Aguilar-Parra J. M. Padilla-Góngora D. López-Liria R. (2019). Organisational climate, role stress, and public employees’ job satisfaction. International Journal of Environmental Research and Public Health, 16(10), 1–12. 10.3390/ijerph16101792 PMC6572401 31117168

[bibr42-00910260211073154] Peters P. Poutsma E. Heijden B. I. J. M. V. d. Bakker A. B. Bruijn T. d. (2014). Enjoying new ways to work: An HRM-Process approach to study flow. Human Resource Management, 53(2), 271–290.

[bibr43-00910260211073154] Petrovsky N. Ritz A. (2014). Public service motivation and performance: A critical perspective. Evidence-Based HRM, 2(1), 57–79. 10.1108/EBHRM-07-2013-0020

[bibr44-00910260211073154] Podsakoff P. M. MacKenzie S. B. Lee J.-Y. Podsakoff N. P. (2003). Common method biases in behavioral research: A critical review of the literature and recommended remedies. Journal of Applied Psychology, 88(5), 879–903. 14516251 10.1037/0021-9010.88.5.879

[bibr45-00910260211073154] Renard K. Cornu F. Emery Y. Giauque D. (2021). The impact of new ways of working on organizations and employees: A systematic review of literature. Administrative Sciences, 11(2), 38.

[bibr46-00910260211073154] Saba T. Cachat-Rosset G. (2020). COVID—19 et télétravail—un remède universel ou une solution ponctuelle. Québec et comparaison internationale. Chaire BMO—Diversité et gouvernance. Université de Montréal. https://www.docdroid.com/AqP0qVn/covid-19-et-teletravail-un-remede-universel-ou-une-solution-ponctuelle-pdf

[bibr47-00910260211073154] Sardeshmukh S. R. Sharma D. Golden T. D. (2012). Impact of telework on exhaustion and job engagement: A job demands and job resources model. New Technology, Work and Employment, 27(3), 193–207. 10.1111/j.1468-005X.2012.00284.x

[bibr48-00910260211073154] Schaufeli W. B. Bakker A. B. (2004). Job demands, job resources, and their relationship with burnout and engagement: A multi-sample study. Journal of Organizational Behavior, 25, 293–315.

[bibr49-00910260211073154] Schaufeli W. B. Leiter M. P. Kalimo R. (1995). The general burnout questionnaire: Cross-national development and validation. Paper presented at the APA/NIOSH Work, Stress and Health ‘95: Creating Healthier Workplaces, Washington, DC, United States.

[bibr50-00910260211073154] Seppälä P. Mauno S. Feldt T. Hakanen J. Kinnunen U. Tolvanen A. Schaufeli W. (2008). The construct validity of the Utrecht work engagement scale: Multisample and longitudinal evidence. Journal of Happiness Studies, 10(4), 459–481.

[bibr51-00910260211073154] ten Brummelhuis L. L. Bakker A. B. Hetland J. Keulemans L. (2012). Do new ways of working foster work engagement? Psicothema, 24(1), 113–120. 22269373

[bibr52-00910260211073154] Thompson C. A. Beauvais L. L. Lyness K. S. (1999). When work–family benefits are not enough: The influence of work–family culture on benefit utilization, organizational attachment, and work–family conflict. Journal of Vocational Behavior, 54(3), 392–415.

[bibr53-00910260211073154] Van den Broeck A. Vansteenkiste M. De Witte H. Lens W. (2008). Explaining the relationships between job characteristics, burnout, and engagement: The role of basic psychological need satisfaction. Work and Stress, 22(3), 277–294.

[bibr54-00910260211073154] van der Lippe T. Lippényi Z. (2020a). Beyond formal access: Organizational context, working from home, and work–family conflict of men and women in European workplaces. Social Indicators Research, 151, 383–402. 10.1007/s11205-018-1993-1 33029037 PMC7505867

[bibr55-00910260211073154] van der Lippe T. Lippényi Z. (2020b). Co-workers working from home and individual and team performance. New Technology, Work and Employment, 35(1), 60–79. 32214593 10.1111/ntwe.12153 PMC7079547

[bibr56-00910260211073154] van der Voordt T. J. M. (2003). Productivity and employee satisfaction in flexible workplaces. Journal of Corporate Real Estate, 6(2), 133-148.

[bibr57-00910260211073154] Van De Voorde K. Paauwe J. Van Veldhoven M. (2012). Employee well-being and the hrm–organizational performance relationship: A review of quantitative studies. International Journal of Management Reviews, 14(4), 391–407.

[bibr58-00910260211073154] Van Steenbergen E. F. van der Ven C. Peeters M. C. W. Taris T. W. (2017). Transitioning towards new ways of working: Do job demands, job resources, burnout, and engagement change? Psychological Reports, 121(4), 736–766. 29298562 10.1177/0033294117740134

[bibr59-00910260211073154] Xanthopoulou D. Bakker A. B. Kantas A. Demerouti E. (2012). Measuring burnout and work engagement: Factor structure, invariance, and latent mean differences across Greece and the Netherlands. International Journal of Business Science & Applied Management, 7(2), 40–52.

[bibr60-00910260211073154] Wang B. Liu Y. Qian J. Parker S. K. (2021). Achieving effective remote working during the COVID-19 pandemic: A work design perspective. Applied Psychology, 70(1), 16–59. 33230359 10.1111/apps.12290 PMC7675760

[bibr61-00910260211073154] Wood J. Oh J. Park J. Kim W. (2020). The relationship between work engagement and work–life balance in organizations: A review of the empirical research. Human Resource Development Review, 19(3), 240–262.

[bibr62-00910260211073154] Wood S. Van Veldhoven M. Croon M. de Menezes L. M. (2012). Enriched job design, high involvement management and organizational performance: The mediating roles of job satisfaction and well-being. Human Relations, 65(4), 419–445.

